# Bazedoxifene as a novel strategy for treatment of pancreatic and gastric adenocarcinoma

**Published:** 2019-05-07

**Authors:** Claudia Burkhardt, Leo Bühler, Matthieu Tihy, Philippe Morel, Michel Forni

**Affiliations:** ^1^ Service de chirurgie viscérale, Département de chirurgie, Hôpitaux Universitaires de Genève, 1211 Genève, Switzerland; ^2^ Département diagnostique Service de pathologie clinique, Hôpitaux Universitaires de Genève, 1211 Genève, Switzerland; ^3^ Clinique de Carouge, Réseau la Tour, Avenue Cardinal Mermillod 1, 1227 Carouge, Switzerland

**Keywords:** cytokine receptor GP130, gastric adenocarcinoma, pancreatic adenocarcinoma, selective estrogene receptor modulator, STAT transcription factors

## Abstract

Experimental studies have shown that the IL6/GP130/STAT3 pathway is involved in pancreatic cancer tumorigenesis and progression as well as in the development of other tumors. Bazedoxifene, a selective estrogene receptor modulator clinically available for the treatment of osteoporosis, has been shown to be an effective GP130/STAT3 signaling inhibitor through in vitro and small animal studies. Our aim was to investigate the effect of bazedoxifene on tumor progression in patients with advanced pancreatic and gastric tumors.

We analyzed the data of 7 patients (5 suffering from pancreatic and 2 from gastric adenocarcinoma), with locally advanced and/or metastatic disease, median age 73 years old (range 48 – 86 years). Bazedoxifene was given orally at a dose of 20 mg per day for a median duration of 9 months (range 5 – 14 months). Two patients received bazedoxifene as monotherapy, 5 patients were under concomitant chemotherapy.

Results showed tumor marker reduction in 5 patients, stable disease on CT in 5 patients and metabolic regression on PET-CT in 3 patients. Weight was gained in 4 patients. Two patients developed deep vein thrombosis and one pulmonary embolism, the treatment was otherwise well tolerated. An immunhistochemical study of pSTAT3 was performed in 6 patients, out of which 3 were positive.

Our preliminary data indicate that bazedoxifene is a potential new therapeutic option for pancreatic and gastric cancer therapy, safe to use and at low cost. It might be administrated at an early stage with current strategies. Based on these preliminary results, we will initiate a prospective clinical study.

## INTRODUCTION

Pancreatic cancer represents a significant cause of morbidity and mortality. 80% of patients present with unresectable disease at diagnosis, and amongst the patients undergoing resection, 80% will develop local recurrence and/or distant metastases and die within 5 years [1].

For localised resectable pancreatic adenocarcinoma, current recommendations include surgical resection followed by 6 months adjuvant therapy. Options are for example Fluoropyrimidine or Gemcitabine with concurrent radiotherapy.

The treatment strategies in locally advanced tumors may depend on whether the disease is resectable, unresectable or borderline resectable. Neoadjuvant therapy can be considered. In metastatic disease, current options include for example chemotherapy with Folfirinox or Gemcitabine plus Abraxane [2]. Recommendations may also vary according to the patient’s performance status.

Gastric cancer is the 5^th^ most frequently diagnosed cancer and is also a leading cause of death from cancer, being often diagnosed at an advanced stage [3]. The preferred therapeutic approach for localised gastric cancer is perioperative chemotherapy or postoperative chemotherapy plus chemoradiation. FLOT (5-Fluorouracil, Leucovorin, Oxaliplatin and Docetaxel) as a perioperative chemotherapy has been linked to an improved outcome in patients with resectable gastric and gastroesophageal junction adenocarcinoma [4]. For advanced and metastatic disease, chemotherapy can provide palliation of symptoms and improved survival and quality of life [3].

Both pancreatic and gastric cancers are responsible for high morbidity and mortality, with limited treatment possibilities. There is a need for new treatment modalities, in particular for patients with unresectable or metastatic disease. The discovery of signaling pathways involved in tumorigenesis may offer new targets for cancer therapy. New therapies are in development in advance gastric tumors targeting the Programmed Death receptor 1 (PD-1) with interaction between cancer cells and stroma (Nivolumab) but disruption of stromal molecular and cellular compents in pancreatic cancer are still under investigation [5] [6].

Studies have shown that the pro-inflammatory cytokine IL6 is involved in pancreatic cancer development and progression, through activation of the GP130/JAK

STAT3 cascade [7] [8]. IL6 is elevated in the serum of pancreatic cancer patients [9]. Inhibiting the IL6/GP130/STAT3 pathway might therefore be a new therapeutic option for pancreatic cancer.

Bazedoxifene, a selective estrogen receptor modulator, has been proven to be an effective GP130/STAT3 signaling inhibitor, inhibiting growth and migration of pancreatic cancer cells in animal studies [9]. It may be a potential therapeutic option for pancreatic cancer therapy. It is already available on the market for the prevention and treatment of osteoporosis. Recommendations are to take a pill every day, usually 20 mg, orally.

## RESULTS

After a median follow-up of 9 months, results showed tumor marker reduction in 5 patients. 5 patients presented stable disease on CT and 3 presented metabolic regression on PET-CT (including 0 and 1 patients with bazedoxifene monotherapy, respectively). Weight was gained in 4 patients. Results specific to pancreatic and gastric adenocarcinoma are shown in Table 1.

**Table 1:** Results after median follow-up of 9 months. Results in percentages are shown in parentheses

	Pancreatic adenocarcinoma (out of 5 patients)	Gastric adenocarcinoma (out of 2 patients)	Overall (out of 7 patients)
Median follow-up (months)	9	10.5	9
Tumor marker reduction (no. of patients)	4 (80%)	1 (50%)	5 (71 %)
Stable disease on CT (no. of patients)	3 (60%)	2 (100%)	5 (71 %)
Metabolic regression PET-CT (no. of patients)	3 (60%)	0 (0%)	3 (43 %)
Weight gain (no. of patients)	4 (80%)	0 (0%)	4 (57 %)

One patient in particular showed complete remission. This patient presented initially with locally advanced unresectable pancreatic cancer. He showed tumor progression after chemotherapy (3 cycles of Gemcitabine, Oxaliplatine and 3 cycles of Gemcitabine, Abraxane). He subsequently received palliative radiotherapy (35 Gy in 5 fractions). Bazedoxifene was initiated two months before radiotherapy. After 11 months of bazedoxifene monotherapy, CA 19-9 initially dropped by 30% (from 25 after 2 months to 18 U/mL after 11 months of bazedoxifene). After 14 months of bazedoxifene, CA 19-9 value remained in the negative range.

Initial hypermetabolic lesion became inactive on PET-CT and tumor mass on PET-CT was reduced in size by 7 % (from 42 mm x 26 mm to 41 mm x 25 mm). His quality of life was greatly improved, allowing him to go back to work full time.

**Figure 1:**
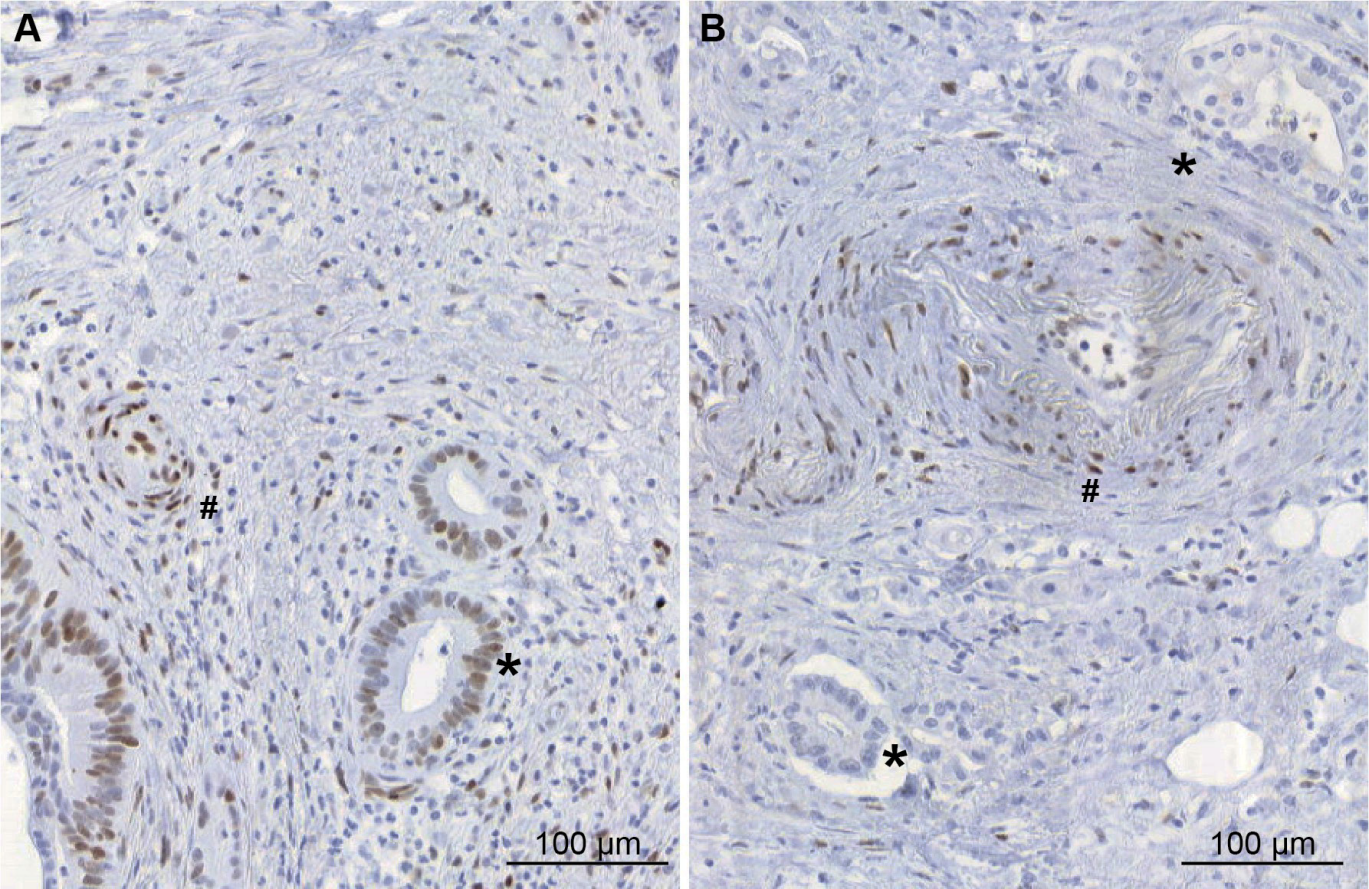
Immunohistochemical study of pSTAT3 expression in pancreatic adenocarcinoma tissues **(A)** example of pSTAT3 positive expression in tumour cells (^*^) **(B)** example of pancreatic adenocarcinoma tissue without pSTAT3 expression in tumour cells (^*^). In both pictures, endothelial cells express pSTAT3 (#).

The immunhohistochemical study using phosphorylated STAT3 (pSTAT3) was positive in 3 out of 6 patients (including 2 patients with pancreatic and 1 with gastric adenocarcinoma). Examples of a positive and a negative sample for pSTAT3 in pancreatic adenocarcinoma tissue of 2 patients are shown in Figure 1.

During the follow-up, 2 patients developed deep thrombosis on the legs, among them one pulmonary embolism, responding well to anticoagulants. Venous thromboembolism is listed as an occasional (less than 1/100, more than 1/1000) in the Swiss Compendium. However, it can also be a complication of advanced stage cancer, which the 2 patients presented.

One patient presented repeated vomiting and gastroparesis, motivating temporary discontinuation of bazedoxifene, otherwise the treatment was well tolerated (even under radiotherapy) easily available and at low cost.

There was no death during the median period of 9 months of follow-up. For comparison, overall one-year survival is of 20% and 41% in patients with pancreatic and gastric cancer, respectively [10] [11]. Patients with unresectable, locally advanced disease, have a median survival of 6 to 11 months. Patients with metastatic disease have a median survival of only 2-6 months.

## DISCUSSION

This is the first study to explore the effect of bazedoxifene on pancreatic and gastric adenocarcinoma in patients. It is already available on the market, safe to use and represents a low cost.

The analysed mechanism was the inhibition of the IL6/GP130/STAT3 cascade. STAT3 has been shown to play a critical role in pancreatic tumorigenesis [12] [13]. Through its phosphorylation in the cytoplasma, STAT3 translocates to the nucleus, promoting the transcription of antiapoptotic and proliferative genes.

Bazedoxifene, a selective estrogen receptor modulator, has been proved to be an effective GP130/STAT3 signaling inhibitor [9]. It has been approved for the treatment of osteoporosis in postmenopausal women. It preserves bone mineral density and reduces total cholesterol levels, without stimulation of the endometrium, ovaries, or breasts [14] [15]. Moreover, it has been shown to block the proliferation of MCF-7 breast cancer cells [16].

Bazedoxifene binds to the GP130 D1 domain, disabling the dimerisation of Il6/IL6Ralpha/GP130. Subsequently, it inhibits STAT3 phosphorylation and transcription induced by IL6, causing apoptosis, blocking cell migration in pancreatic cancer cells and suppressing tumor growth [9]. Bazedoxifene also shows synergism with Paclitaxel or Gemcitabine, further inhibiting pancreatic cancer cell migration and decreasing viability.

Furthermore, various studies have shown the effect of bazedoxifene on other tumors through IL6/GP130 signaling. In head and neck squamous cell carcinoma, bazedoxifene inhibits cell proliferation, migration and colony formation, it reduces chemo and radioresistance and enhances the effects of cisplatin and radiation treatment with no added systemic toxicity [17]. In rhabdomyosarcoma cells, bazedoxifene induces apoptosis, inhibits cell invasion, angiogenesis, colony formation and suppresses tumor growth [18] [19].

The number of patients included in our study was small, however, the results were promising. After a median of 9 months follow-up under bazedoxifene monotherapy and/or combined with chemotherapy, patients showed tumor marker reduction, metabolic regression, tumor size reduction and improved quality of life.

As all of the patients were still alive at the end of the follow-up, the overall survival length was not available, but the median survival was at least of 9 months in patients with locally advanced and/or metastatic disease, which is higher than the median survival described in the literature. However, 5 out of the 7 patients were under concomitant chemotherapy so the longer survival and tumor size reduction cannot be attributable to Bazedoxifene alone.

Our immunohistochemical study, on the other hand, showed mixted results as pSTAT3 was expressed in only in 3 out of 6 patients. This suggest that some cancers progress through the activation of GP130/STAT3 cascade but other cancers develop through other pathways.

Therefore, through this preliminary study, we bring attention to bazedoxifene as a new potential therapeutic option for pancreatic cancer therapy as well as for other tumors, in addition with current strategies.

## MATERIALS AND METHODS

We conducted a retrospective study on 7 patients, 5 suffering from pancreatic and 2 from gastric adenocarcinoma. These patients were all at risk for osteoporosis, 3 of them were osteopenic (T score between -1.0 and -2.5 on bone mineralometry). Median age was 73 years old (range 48 – 86 years). Bazedoxifene was prescribed according to its original indication, which is prevention and treatment of osteoporosis, in patients who could also benefit from its effect on tumor growth and progression.

All of the patients were followed by the same oncology office in Geneva. 2 patients presented locally advanced disease without metastasis and 4 patients presented metastatic disease. One patient with resectable pancreatic adenocarcinoma was included. 3 patients had undergone surgical resection, 1 of which with positive resection margin (R1).

Bazedoxifene was given orally at a dose of 20 mg per day for a median duration of 9 months (range 5 – 14 months). Two patients received bazedoxifene as monotherapy, 5 patients were under concomitant chemotherapy (Gemcitabine and Nab-Paclitaxel weekly).

Patient written consent was obtained and the study was authorised by the Swiss Ethics committee, in accordance with the Declaration of Helsinki.

The primary objective was to investigate the effect of bazedoxifene on tumor progression through study of CT, PET-CT and tumor marker results. Its effect on weight gain was also assessed. The secondary objective was to study the molecular mechanisms of the GP130/STAT3 pathway in pancreatic and gastric adenocarcinoma through an immunohistochemical study. For this purpose, cancerous tissue sections of 6 out of 7 patients were incubated with an anti-STAT3-p-tyr705 monoclonal antibody. These samples were taken prior to the administration of Bazedoxifene. The immunohistochemical study was not possible in one of the patients due to insufficient tissue available.

## CONCLUSIONS

Our preliminary data indicate that bazedoxifene could be a potential new therapeutic option for pancreatic and gastric cancer therapy, through inhibition of GP130 signaling. It is already clinically available, is safe to use and at low cost. It might be administrated at an early stage concomitantly with current strategies.

The patients included in this study, presenting with a locally advanced or metabolic disease, showed tumor marker and size reduction, as well as an improved quality of life under bazedoxifene. Overall survival rates weren’t available as there was no death during the median follow-up of 9 months.

Based on these preliminary results, we will initiate a large-scale, prospective, randomised clinical study.

## Abbreviations

IL6: Interleukin 6
GP130: Glycoprotein 130
STAT: Signal Transducer and Activator of Transcription
CT: Computed Tomography
PET: Positron Emission Tomography
FLOT: 5-Fluorouracil, Leucovorin, Oxaliplatin and Docetaxel
PD1: Programmed Death Receptor 1
JAK: Janus kinase.
